# Supplementary material for: Alcohol consumption in the G7 countries (1960–2021). Permanent versus transitory shocks
Source: PLoS One. 2024 Dec 4;19(12):e0314877. doi: 10.1371/journal.pone.0314877 (PMC11616831; doi:10.1371/journal.pone.0314877)
Supplement: S1 File — (PDF) [file pone.0314877.s001.pdf]

Alcohol Consumption G7

Country	1960	1961	1962	1963	1964	1965	1966	1967	1968	1969	1970	1971	1972	1973	1974	1975	1976	1977	1978	1979	1980
**Canada**	7	7.1	7.3	7.5	7.6	7.8	8.1	8.3	8.3	8.5	8.8	9.4	9.9	10.4	10.8	10.9	10.9	10.9	10.9	10.9	10.7
**France**											23.2	22.9	22.6	22.8	22.5	22.5	22.2	22.1	21.1	20.6	20.1
**Germany**		11	12.1	12.9	13.7	14	13.6	13.5	14	15	15.5	16.5	16.3	16.7	15.9	16.8	17.2	16.4	16.3	16.7	16.5
**Italy**			19.4	18.2	17.8	18.4	18.7	19	19.7	19.7	19.7	19.6	19.5	19.9	19.7	18.3	17.6	17	16.5	16.5	16.7
**Japan**					5.2	5.3	5.3	5.4	5.5	5.8	6.1	6.1	6.2	6.5	6.6	6.7	6.7	7	7	7.2	7.1
**United Kingdom**		7.1	7.1	7.2	7.5	7.4	7.6	7.7	7.9	8.1	7.1	7.4	7.8	8.7	9.1	9	9.3	8.8	9.4	9.8	9.4
**United States**	7.8	7.8	8	8.1	8.4	8.6	8.8	9	9.3	9.5	9.5	9.8	9.6	9.8	10	10.1	10.1	9.8	10.1	10.2	10.4
	**1960**	**1961**	**1962**	**1963**	**1964**	**1965**	**1966**	**1967**	**1968**	**1969**	**1970**	**1971**	**1972**	**1973**	**1974**	**1975**	**1976**	**1977**	**1978**	**1979**	**1980**

Country	1981	1982	1983	1984	1985	1986	1987	1988	1989	1990	1991	1992	1993	1994	1995	1996	1997	1998	1999	2000
**Canada**	10.8	10.5	10.2	9.9	9.8	9.6	9.6	9.4	8.8	8.5	8.2	7.8	7.5	7.4	7.4	7.4	7.2	7.3	7.5	7.6
**France**	19.5	19.4	18.8	17.9	17.6	17.1	16.8	16.8	17	15.4	15.4	15.4	14.7	14.6	14.5	14.5	14.3	14.5	14.4	13.9
**Germany**	16	15.7	15.8	15.1	15.1	14.7	14.8	14.6	14.6	14.9	13.9	13.8	13.5	13.4	13.4	13.1	13	12.7	12.8	12.9
**Italy**	15.1	14.5	14.6	14.1	13.2	12.4	11.8	11.4	11	11	10.8	10.6	10.3	10.1	9.6	9.1	9.1	9	8.9	9.8
**Japan**	7.2	7.3	7.5	7.4	7.3	7.5	7.9	8.3	8.6	9.2	8.9	8.9	9.2	9.1	8.9	9	8.8	8.7	8.8	8.6
**United Kingdom**	9.1	8.8	9.1	9.2	9.3	9.3	9.5	9.8	9.8	9.8	9.4	9.3	9.3	9.6	9.3	9.8	10	9.8	10.3	10.4
**United States**	10.4	10.3	10.2	10	9.9	9.8	9.7	9.4	9.2	9.3	8.7	8.7	8.4	8.3	8.1	8.2	8.1	8.1	8.2	8.3
	**1981**	**1982**	**1983**	**1984**	**1985**	**1986**	**1987**	**1988**	**1989**	**1990**	**1991**	**1992**	**1993**	**1994**	**1995**	**1996**	**1997**	**1998**	**1999**	**2000**

Country	2001	2002	2003	2004	2005	2006	2007	2008	2009	2010	2011	2012	2013	2014	2015	2016	2017	2018	2019	2020	2021	MAX	MIN	MEAN	DESV
**Canada**	7.6	7.7	7.7	7.8	8	8.2	8.4	8.4	8.4	8.4	8.2	8.3	8.2	8	8	8.1	8.2	8.2	8	8.1	8.3	10.9	7	8.62258065	1.19031878
**France**	14.1	13.9	13.4	13.1	12.9	13.1	12.9	12.5	12.6	12.3	12.4	12.2	11.6	12	11.9	11.7	11.7	11.6	11.4	10.4	10.5	23.2	10.4	15.8326923	3.91471438
**Germany**	12.5	12.3	11.9	11.8	11.7	11.8	11.5	11.4	11.2	11.6	11.3	11.3	11.1	11.1	11.1	11	10.9	10.9	10.6			17.2	10.6	13.5830508	1.9718845
**Italy**	9.7	9.3	9.3	9	8.7	8.4	8.4	8	7.3	7	7	7.5	7.4	7.6	7.1	7.1	7.4	7.8	7.7			19.9	7	12.5862069	4.62788832
**Japan**	8.6	8.4	8.4	8.2	8.5	1.9	7.7	7.5	7.4	7.3	7.3	7.2	7.4	7.1	7.2	7.2	7.2	7.2	7.1	6.7	6.6	9.2	1.9	7.32586207	1.30243501
**United Kingdom**	10.8	11.1	11.3	11.6	11.4	11	11.1	10.8	10.1	10.1	9.9	9.6	9.4	9.4	9.5	9.5	9.7	9.8	9.7	9.7	10	11.6	7.1	9.34098361	1.12877176
**United States**	8.3	8.3	8.4	8.5	8.5	8.6	8.7	8.7	8.7	8.6	8.7	8.9	8.8	8.8	8.8	8.9	8.9	8.9	9	9.2	9.5	10.4	7.8	9.02741935	0.73130679
	**2001**	**2002**	**2003**	**2004**	**2005**	**2006**	**2007**	**2008**	**2009**	**2010**	**2011**	**2012**	**2013**	**2014**	**2015**	**2016**	**2017**	**2018**	**2019**	**2020**	**2021**				

The gaps in yellow represent unavailable data, not that they have been forgotten.

Source: OECD. (2023). Alcohol consumption. https://doi.org/10.1787/e6895909-en
